# Chitosan-Based Self-Healing Hydrogel: From Fabrication to Biomedical Application

**DOI:** 10.3390/polym15183768

**Published:** 2023-09-14

**Authors:** Siyu Pan, Chongyu Zhu, Yuwei Wu, Lei Tao

**Affiliations:** 1 The Key Laboratory of Bioorganic Phosphorus Chemistry & Chemical Biology, Ministry of Education, Department of Chemistry, Tsinghua University, Beijing 100084, China; psy21@mails.tsinghua.edu.cn; 2 Key Laboratory of Science and Technology of Eco-Textile, Ministry of Education, College of Chemistry and Chemical Engineering, Donghua University, Shanghai 201620, China; czhu@dhu.edu.cn; 3 The Second Dental Center, Peking University School and Hospital of Stomatology, Beijing 100101, China

**Keywords:** chitosan, self-healing, hydrogel, fabrication, tissue engineering, customized drug delivery, smart biosensors, three/four dimensional (3D/4D) printing

## Abstract

Biocompatible self-healing hydrogels are new-generation smart soft materials that hold great promise in biomedical fields. Chitosan-based self-healing hydrogels, mainly prepared via dynamic imine bonds, have attracted broad attention due to their mild preparation conditions, excellent biocompatibility, and self-recovery ability under a physiological environment. In this review, we present a comprehensive overview of the design and fabrication of chitosan-based self-healing hydrogels, and summarize their biomedical applications in tissue regeneration, customized drug delivery, smart biosensors, and three/four dimensional (3D/4D) printing. Finally, we will discuss the challenges and future perspectives for the development of chitosan-based self-healing hydrogels in the biomedical field.

## 1. Introduction

Hydrogels are soft materials that have 3D networks holding a high water content. Their unique structures have similarity to the extracellular matrix (ECM), and are beneficial for cell metabolism and tissue growth, demonstrating their potential in biomedical applications [1,2,3]. Inspired by the spontaneous wound repair of organisms, hydrogels with self-healing properties, known as self-healing hydrogels, have been developed over recent decades [4,5,6,7]. Upon being mechanically fractured, these hydrogels will reassemble their fragments, and restore their integrity and mechanical properties. Hence, they can be formulated into injectable form for various biomedical applications, such as cell or drug therapy. Compared to traditional two-component injectable hydrogels that require a gelling process during injection, these self-healing hydrogels can be injected after gel formation. Therefore, there are no concerns about unreacted precursors, needle blocking due to the fast gelling process, or leakage of cargoes during injection. More importantly, these self-healing hydrogels can transform into new geometric shapes to adapt to their surroundings at the injection site [8,9,10].

Chitosan is a cationic polymer obtained by partial deacetylation of chitin, which is a natural polysaccharide in fungi, the exoskeleton of crustaceans and insects [11]. Possessing excellent biocompatibility, biodegradability, antibacterial ability and cell affinity, chitosan is an ideal candidate for the fabrication of a self-healing hydrogel for biomedical applications [12]. The key to installing a spontaneous self-healing property onto chitosan-based hydrogels is to construct a dynamic physical or chemical crosslinked 3D network. Thanks to the abundant hydroxyl and amine groups on the chitosan chain, both noncovalent interactions (such as hydrogen bonding [13], electrostatic interactions [14,15], hydrophobic interactions [16], etc.) and dynamic covalent bonds (i.e., borate ester [17], acyhydrazone [18], imine [19] and disulfide bonds [20]) have been introduced to generate dynamic 3D frameworks in chitosan-based hydrogels with a self-healing property. Among these, imine bonds can be formed and are reversible under a physiological environment, leading to facile preparation, as well as broad application in biomedical fields.

In this review, we summarize the design and fabrication of chitosan-based self-healing hydrogels. Their applications in tissue regeneration, customized drug delivery, smart biosensors and 3D/4D printing are further discussed.

## 2. Design and Fabrication

The key to the preparation of chitosan-based self-healing hydrogels is to build up a hydrogel network with dynamic crosslinking points. Such crosslinking points can be either generated from physical interactions or dynamic chemical bonds [12,21]. Thanks to the plentiful amino groups on the chitosan structure, one simple approach to construct dynamic crosslinking points is to develop suitable crosslinkers with aldehyde groups [22]. These aldehyde groups can spontaneously react with the amino side chains on chitosan under physiological conditions, forming imine bonds as dynamic crosslinking points, resulting in hydrogels with self-healing property.

However, the choice of crosslinkers is crucial to the final performance of these chitosan-based self-healing hydrogels. The varied reactivity difference when comparing the aldehyde structures will change the self-healing speed and gelling rate of the hydrogels. Meanwhile, the molecular weight, the geometry and chain flexibility of these crosslinkers will have a notable impact on the mechanical properties of these hydrogels. Moreover, the biocompatibility of these crosslinkers should be considered. For example, although small molecular aldehydes such as glutaraldehyde are good crosslinkers for forming chitosan-based self-healing hydrogels, their neurotoxicity is still a barrier to their biological applications [23,24]. Introducing aldehyde groups into biocompatible polymers, such as polyethylene glycol (PEG) and natural polysaccharide, has proven to be a useful strategy to enhance the bio-safety of these crosslinkers, a promising path for chitosan-based self-healing hydrogels in biomedical use.

**Figure 1:**
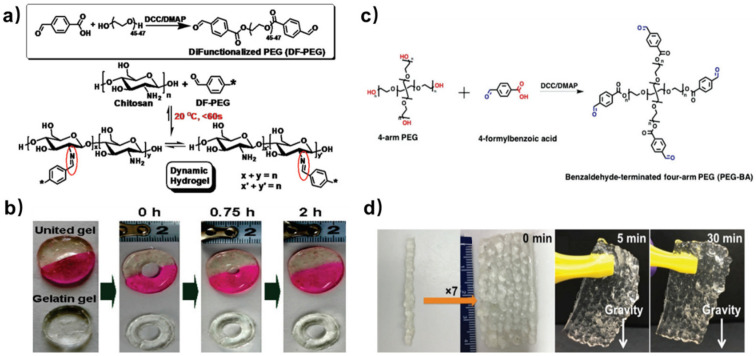
(**a**) Schematic illustration of the dynamic hydrogel. * denotes the rest of DF-PEG polymer chain. (**b**) Self-healing process of the hydrogel. Reprinted with permission from ref. [25]. Copyright 2011, American Chemical Society. (**c**) Synthesis of PEG-BA. (**d**) Macroscopic recovery process of CMC/PEG-BA hydrogel. Reprinted with permission from ref. [26]. Copyright 2016, Wiley-VCH.

In 2011, Zhang et al. prepared a dibenzaldehyde-terminated telechelic PEG crosslinker (DF-PEG) by one-step esterification between 4-formylbenzoic acid and hydroxyl-terminated PEG [25]. By mixing the DF–PEG solution with a chitosan solution, a dynamic hydrogel was formed within 60 s at around 20 °C (Figure 1a). In such mild conditions, the aldehyde groups on the DF–PEG reacted with the amino groups along the chitosan chain to form dynamic imine bonds, serving as crosslinking points in the hydrogel. To evaluate the self-healing ability of this hydrogel, the authors prepared a hydrogel with a hole in the middle and half-dyed red, observing its shape evolution over time (Figure 1b). With time, the hole in the hydrogel became smaller, and eventually disappeared after 2 h. The color boundary in this hydrogel also blurred as the dyes migrated along with the reconstruction of the hydrogel network. On the contrary, a gelatin gel punched with a hole in the middle showed no signs of recovery. In addition to self-healing performance, the dynamic imine bonds also offer the hydrogel a stimuli–response property against several biomedical stimuli, including pH, amino acids and vitamin B6 derivatives. Hence, this hydrogel can act as a drug carrier for water soluble bioactive molecules like lysozyme, realizing controllable release under physiological conditions.

Compared to linear PEG, four-arm PEG behaves as a group of impenetrable space-filled spheres, with less physical entanglements in aqueous conditions. Hence, aldehyde crosslinkers derived from four-arm PEG are expected to form stronger hydrogels with chitosan, giving higher fracture resistance compared to equivalent two-arm based networks with the same average crosslink density. Huang et al. reported a benzaldehyde-terminated telechelic four-armed PEG (PEG-BA, Figure 1c) [26]. The addition of PEG-BA into carboxymethyl chitosan (CMC) solutions produced a dynamic hydrogel through the imine bonds. Compared to the chitosan/DF-PEG hydrogel (~1000 Pa), the CMC/PEG-BA hydrogel had a higher storage modulus (~3000 Pa) owing to the four-arm PEG crosslinkers. Inheriting the self-healing property from the dynamic imine crosslinking points, this hydrogel can be injected into strips and grabbed into an integrated piece at room temperature (Figure 1d). Moreover, the healing process of CMC/PEG-BA hydrogel only takes 5 min, much faster than chitosan/DF-PEG hydrogel (2h). This is because fewer imine bonds are required to re-form with the same network density in CMC/PEG-BA hydrogel than that in chitosan/DF-PEG hydrogel. Owing to the enhanced mechanical property, chitosan-based self-healing hydrogel formed by PEG-BA has become a candidate material for specific biomedical fields, such as meniscus tissue engineering and wound healing.

**Figure 2:**
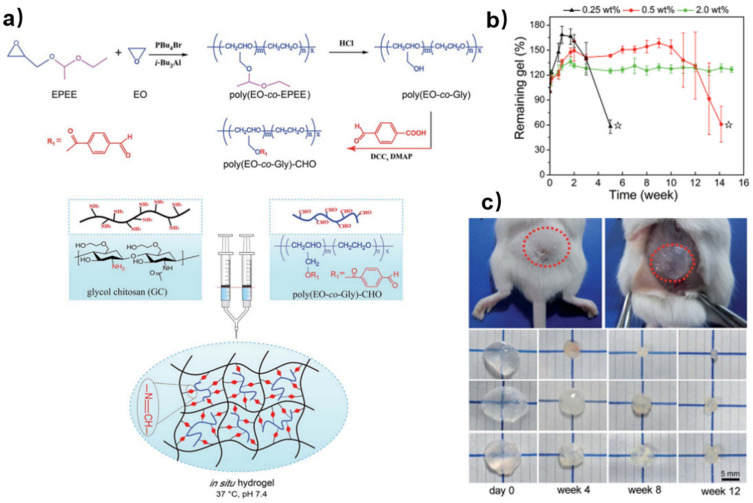
(**a**) Schematic representation of the formation of self-healing hydrogels cross-linked by Schiff’s base reaction between GC and poly(EO-co-Gly)-CHO. (**b**) In vitro degradation of GC/poly(EO-co-Gly) hydrogels prepared with different concentrations of poly(EO-co-Gly)-CHO in PBS containing 1 mg/mL lysozyme at 37 °C. (**c**) In vivo degradation of the hydrogels. Reprinted with permission from ref. [27]. Copyright 2015, Royal Society of Chemistry.

Multi-aldehyde functionalized PEG can further increase the possible crosslinking sites and provide better tunability of gelation time and degradation behavior. Cao and co-workers reported an injectable self-healing hydrogel by crosslinking multi-benzaldehyde PEG (poly(EO-co-Gly)-CHO) and glycol chitosan (GC) (Figure 2a) [27]. Firstly, poly(EO-co-EPEE) was synthesized by anionic copolymerization of EPEE and EO. After deprotection, benzaldehyde groups were introduced into the polymer backbone by esterification between poly(EO-co-Gly) and 4-formylbenzoic acid. The degradation behavior of the hydrogel plays an important role in tissue engineering. According to the results of hydrogel degradation in vitro (Figure 2b), hydrogels with a higher concentration of crosslinkers had lower water uptake during the first two weeks, because the denser network provided less flexibility for water molecules to come through. The results of hydrogel degradation in vivo agreed well with that in vitro (Figure 2c). When hydrogels with different numbers of crosslinkers were implanted in the backs of mice, the size of the remaining hydrogel with more crosslinkers decreased relatively slowly compared to that with fewer crosslinkers. Therefore, these in situ hydrogels can be applied as implantable biomaterials with tunable degradation behaviors in vivo.

**Figure 3:**
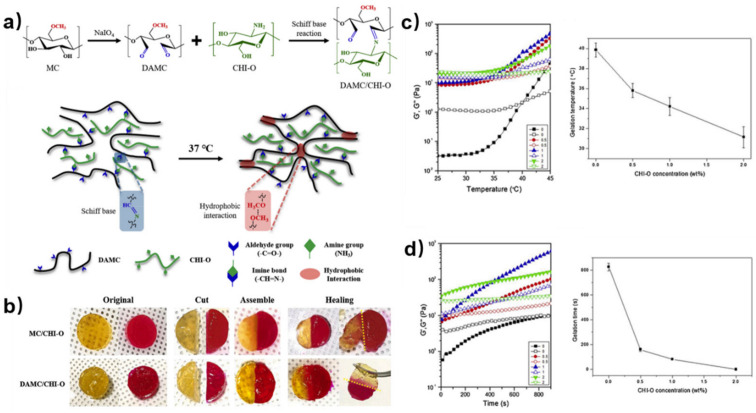
(**a**) Synthesis of DAMC and its reaction with CHI-O to form DAMC/CHI-O hydrogels (top); formation of dual-crosslinked DAMC/CHI-O hydrogel network via covalent imine bonds and hydrophobic interactions at 37 °C (bottom). (**b**) Self-healing behaviors of MC/CHI-O and DAMC/CHI-O. (**c**) Rheological data and the gelation temperature of the DAMC/CHI-O (CHI-O: 0–2 wt%). (**d**) Rheological data and the gelation time of the DAMC/CHI-O (CHI-O: 0–2 wt%). Reprinted with permission from ref. [28]. Copyright 2021, Elsevier Ltd.

Besides synthetic polymers, natural macromolecules are also good precursors as aldehyde crosslinkers. Such examples include sodium alginate, methylcellulose, dextran, et al. Through chemical oxidation, the hydroxy side chains of these natural polysaccharides will partially convert into aldehyde groups, producing crosslinkers with excellent cytocompatibility. In the research conducted by Yeo and co-workers [28], the methylcellulose (MC) was oxidized by NaIO4 to its dialdehyde form (DAMC), which further reacted with the amino groups of the chitosan oligomer (CHI-O) to from imine bonds (Figure 3a). Although the MC without oxidation can also form hydrogels by hydrophobic interactions along the MC chains (MC/CHI-O), introducing a dynamic imine network (DAMC/CHI-O) into the hydrogel will further enhance its self-healing speed (Figure 3b) as well as mechanical performance. The gelation temperature and time are adjustable by different CHI-O concentration. With increased temperature, the hydrogel underwent sol-to-gel transition. The hydrogel with higher CHI-O concentration had lower gelation temperature (Figure 3c) and shorter gelation time (Figure 3d). It is noteworthy that the process of generating aldehyde groups on polysaccharides requires severe oxidation, which may impair the structure of the backbone; thus, finest control of the reaction conditions is needed.

## 3. Biomedical Applications

Self-healing hydrogels are excellent carriers for delivery of various cargoes (cells, bioactive molecules, drugs, et al.) for different requirements [29]. The self-healing hydrogels based on chitosan and aldehyde crosslinkers can achieve adjustable mechanical properties and high biocompatibility, which is important in maintaining the functions of encapsulated cargoes. Chitosan-based self-healing hydrogels with imine bonds are relatively stable in normal physiological conditions (pH~7); however, several stimuli such as changes in pH values [30,31], temperature [32,33] and presence of enzymes [25,34] can disrupt the hydrogel. This property enables controlled release of cargoes, which is valuable in drug delivery and tissue engineering applications. Moreover, chitosan stands out as a promising material for biological sensors owing to its biocompatibility and possible electrical conductivity. The injectability and self-healing ability of chitosan-based hydrogel make it an ideal material for 3D/4D printing. In this section, we will summarize the recent developments on the applications of chitosan-based self-healing hydrogels in tissue regeneration, customized drug delivery, smart biosensors and 3D/4D printing.

### 3.1. Tissue Regeneration

Tissue regeneration is a complex biological process that involves the proliferation and differentiation of cells [35]. For tissue regeneration, methods are required to repair and replace the injured tissues. Cell therapy has served as a direct method to treat diseases by replacing the physiological functions of defected tissues. The key to cell therapy is how to transplant cells to the defected sites and maintain their bioactivity and functions. One possible strategy is to incorporate cells into protective biomaterials. Injectable self-healing chitosan-based hydrogels can encapsulate various cargoes (i.e., cells and bioactive molecules) and deliver them in order to repair tissues at a high concentration without impairing the bioactivity of the cargoes [36]. Thus, chitosan self-healing hydrogels are ideal candidates for tissue regeneration, such as in nerves and skin.

**Figure 4:**
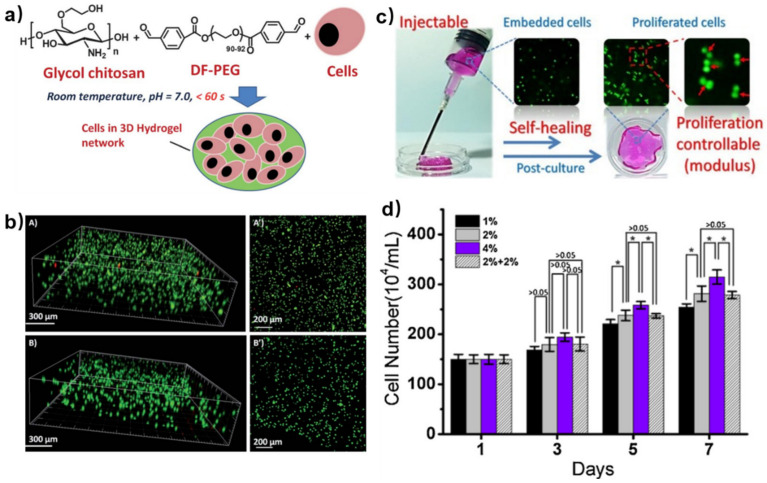
(**a**) Preparation of cells loaded in GC/PEG hydrogels under physiological conditions. (**b**) 3D (**A**,**B**) and z-axis maximum projection (**A**’,**B**’) views of confocal microscopy images. Cell viability (viable cells: green, dead cells: red) and spatial distribution of HeLa cells encapsulated in the hydrogels after 24 h (**A**) and 72 h (**B**). Reprinted with permission from ref. [37]. Copyright 2012, Royal Society of Chemistry. (**c**) Injection of cell-embedded GC/PEG hydrogel and cell proliferation behavior in the hydrogel. (**d**) Cell numbers in a series of hydrogels with different modulus at denoted time after injection. Data represent mean *±* SD (*n* = 3, *: *p* < 0.05). Reprinted with permission from ref. [38]. Copyright 2017, Elsevier Ltd.

For better cell encapsulation, hydrogels need to be biocompatible and have a suitable gelation rate for cells to evenly distribute. Early in 2012, Yang et al. prepared a self-healing hydrogel using DF-PEG and GC to embed cells (Figure 4a) [37]. Before cell encapsulation, the biosafety of gel precursors was evaluated by cell experiments. The cell viability was greater than 80%, and even the concentration of DF-PEG reached 9.0 mg/mL. Thus, the GC/PEG hydrogel had excellent biocompatibility considering that GC is a well-known biocompatible biopolymer. After mixing cell-suspended GC solution with DF-PEG solution in a mild condition, the GC/PEG hydrogel with uniformly-distributed cells was formed, which reached ~97% cell viability after 24 h and still ~87% cell viability after 72 h (Figure 4b). These results indicated that the GC/PEG hydrogel was safe for cell growth as an ECM-like carrier and oxygen supplier. After injection via a needle, there were ~87% live cells in GC/PEG hydrogel and this remained ~85% for 24 h, indicating that most of the cells could tolerate the injection process. Thus, the chitosan-based self-healing hydrogel proved to be a potential cell carrier.

The modulus of hydrogels can affect the behavior of cell proliferation. Li et al. prepared a series of GC/PEG self-healing hydrogels with different storage moduli by simply verifying the amount of DF-PEG [38]. They further investigated the cell proliferation of GC/PEG hydrogels by injecting cell-loaded hydrogels through a syringe to a peri-dish and post-culturing them (Figure 4c). After injection and post-culture, the cell numbers in GC/PEG hydrogels with different moduli increased, indicating cell proliferation behavior in the hydrogels. Moreover, the cell number increased by 70%, 88% and 110% in the soft, medium and stiff hydrogels, which showed the significant positive correlation between the proliferation rate of the loaded cells and the moduli of the hydrogels (Figure 4d).

**Figure 5:**
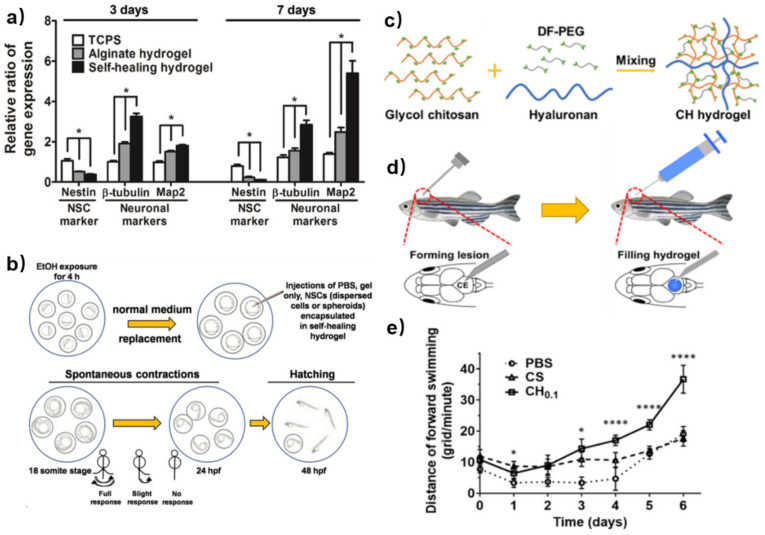
(**a**) The expressions of specific neuronal-related genes (Nestin, *β*-tubulin, Map2) in the TCPS, alginate hydrogel and GC-PEG self-healing hydrogel after 3 d and 7 d. (**b**) Treatment procedures for the central nervous system rescue in zebrafish by the GC-PEG self-healing hydrogel. The zebrafish embryos were exposed to ethanol, resulting in central nervous system deficits. Spontaneous side-to-side contraction of the tail was examined at the 18 somite stage. Reprinted with permission from ref. [40]. Copyright 2015, Wiley-VCH. (**c**) Preparation of Hyaluronan-included GC-PEG hydrogel (CH hydrogel). (**d**) Schematic diagram of the zebrafish model. (**e**) Swimming track of adult zebrafish after the implantation of the hydrogel. * and **** each represent *p* < 0.05 and *p* < 0.0001 between the groups treated with CS and CH hydrogels (*n* = 9). Reprinted with permission from ref. [41]. Copyright 2020, American Chemical Society.

Besides cell proliferation, the mechanical performance of the hydrogel can influence other functions, such as cell differentiation, which is important for tissue regeneration. Neural stem cells (NSCs) are prone to glial differentiation on slightly stiffer materials (≈7*–*10 kPa) and neuronal differentiation in relatively soft materials (≈0.1*–*1 kPa) [39]. Tseng et al. used chitosan-based self-healing hydrogels (GC-PEG hydrogel) with appropriate stiffness (≈1.5 kPa) to heal the impaired central nervous system [40]. According to the RT-PCR tests, cells encapsulated in self-healing hydrogels expressed more neuronal marker genes (*β*-tubulin and Map2) than those on alginate hydrogel and traditional culture plates at 3 d and 7 d. The expression of *β*-tubulin gene (an early neuronal marker) was observed to be down-regulated from 3 d to 7 d, while the expression of Map2 gene (a marker of mature neurons) showed an up-regulation from 3 d to 7 d, particularly in the self-healing hydrogel (Figure 5a). These results indicated that the chitosan-based self-healing hydrogel provided favorable environments for the growth of neuronal cells. Zebrafish embryos after ethanol exposure were used to evaluate the rescue of the neural deficits by different treatments (Figure 5b). Several groups of damaged embryos were injected with the NSCs (dispersed cells or spheroids)-encapsulated GC-PEG hydrogels, dispersed NSC cells and GC-PEG hydrogels only. Among these, the embryos with injection of NSC spheroid-loaded self-healing hydrogels had the highest total of coiling contractions and hatching rates. This indicated that the chitosan-based self-healing hydrogels with embedded NSC spheroid might heal the damaged central nervous system and rescue the impaired nervous functions. The appropriate modulus (≈1.5 kPa) provided adequate porosity for cell metabolism and migration, as well as convenience for the location of the NSC spheroid. The authors made a point that the amino groups on the chitosan backbone might interact with NSCs to induce neuronal differentiation.

In order to enhance the spreading, migration, proliferation and differentiation of embedded NSCs, hyaluronan (HA) was introduced into the chitosan-based self-healing hydrogels. In the research of Liu et al. [41], the HA was incorporated into NSC-embedded self-healing hydrogels to prepare a semi-interpenetrating polymer network (SIPN) (Figure 5c). The SIPN proved to provide a denser nanostructure and a looser microstructure, which promoted the growth of embedded NSCs. The zebrafish traumatic brain injury model was employed to verify the functional recovery (Figure 5d). The swimming behavior of these zebrafish was then recorded. The zebrafish which were injected with HA-containing GC-PEG hydrogels (CH_0.1_) demonstrated the best functional recovery with the largest distance of forward swimming. Injection of GC-PEG hydrogels (CS in Figure 5e) alone exhibited a significant short-term effect, but its impact diminished after 4 days compared to the PBS group, with only minor effects observed (Figure 5e). These results indicated that optimization of the 3D network of chitosan-based hydrogels can probably improve tissue regeneration by changing the cell microenvironment.

**Figure 6:**
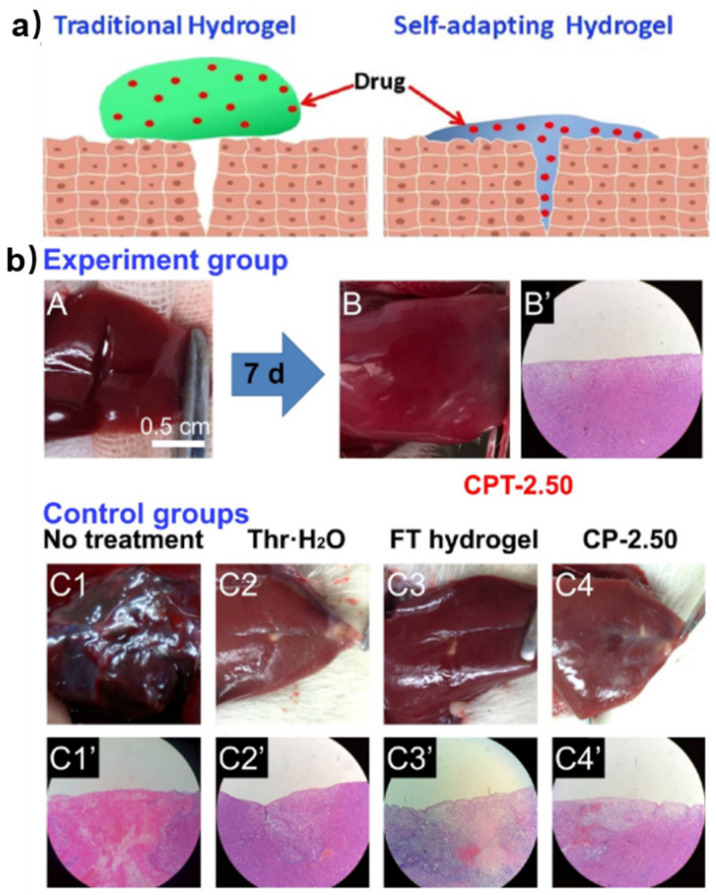
(**a**) Schematic illustration of self-adapting chitosan-based hydrogel. (**b**) Photographs and microscopic evaluation of rat liver laceration with different treatments. (**A**) Cruciate incision wound; wound-healing after 7 days with treatment by (**B**,**B′**) CPT hydrogel; (**C1**,**C1′**) no treatment; (**C2**,**C2′**) thrombin aqueous solution (Thr*·*H_2_O); (**C3**,**C3′**) plurionic hydrogel containing thrombin (FT-hydrogel); (**C4**,**C4′**) chitosan-based hydrogel without thrombin (CP-2.50) (*n* = 6). Reprinted with permission from ref. [42]. Copyright 2018, American Chemical Society.

Apart from encapsulating cells, chitosan-based self-healing hydrogels can also embed bioactive molecules to promote tissue regeneration, such as wound healing. Compared with traditional hydrogels, self-healing hydrogels can adapt to the shape of wounds so that the hydrogel dressing accompanying the medicines can keep complete contact with the wound for better healing. Li et al. reported this kind of self-adapting chitosan-based hydrogel (Figure 6a) [42]. This hydrogel exhibited excellent biocompatibility, self-healing ability and tissue adhesive ability. Moreover, the chitosan-based hydrogel had an adequate degradation rate and thrombin release rate in vitro, which was suitable for long-lasting treatment of wound healing. In in vivo wound-healing tests, a cruciate incision was made using a surgical scalpel on the right lobe of a rat liver, resulting in a greater VI liver laceration (Figure 6b). Then, different treatments were employed including chitosan-based hydrogel containing thrombin (CPT-2.50), thrombin aqueous solution (Thr*·*H_2_O), Plurionic (F127) hydrogel containing thrombin (FT-hydrogel) and chitosan-based hydrogel without thrombin (CP-2.50). During 7 days observation, the damaged liver under the treatment of thrombin-loaded chitosan-based hydrogel was completely healed with excellent tissue regeneration (Figure 6(bA,B)). There was no obvious inflammatory cell infiltration in the regenerated tissue (Figure 6(bB′)) in histological analyses. However, scars on the liver of the other groups were visible (Figure 6(bC1–C4)). Inflammatory cells could still be seen (Figure 6(bC1′–C4′)) and the Thr*·*H_2_O treated group showed relatively slight infection. These results indicated that chitosan-based self-healing hydrogel was an ideal candidate as a carrier for wound-healing because of its physical adhesion to the wound and by using the natural anti-inflammatory chitosan as a gelator. The thrombin can perform better as a bioactive factor when encapsulated in the chitosan-based self-healing hydrogel than in direct use.

**Figure 7:**
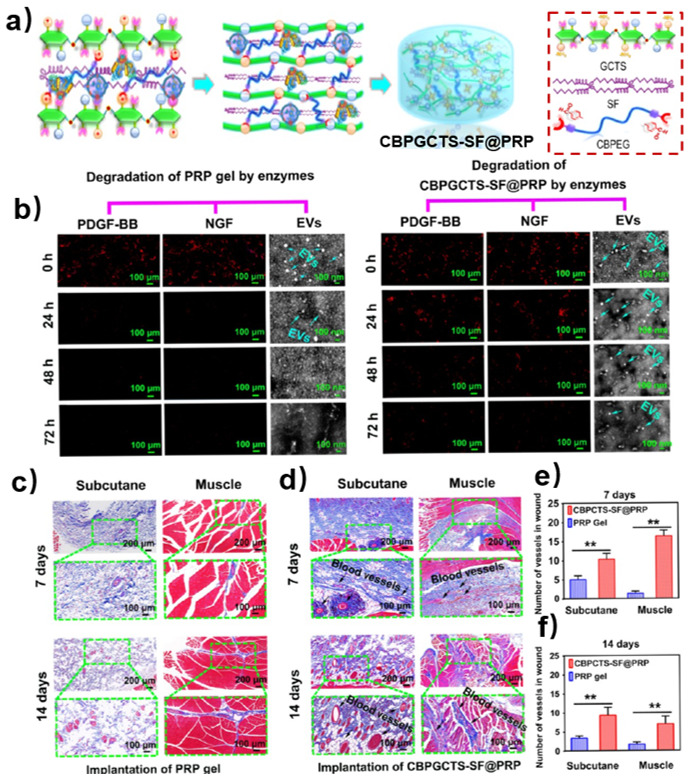
(**a**) Fabrication of PRP-loaded chitosan-based self-healing hydrogel (CBPGCTS) with the addition of biocompatible and enzymatic hydrolysis-resistant silk fibroin (SF) (CBPGCTS-SF@PRP). (**b**) PDGF-BB, NGF, and EVs contents in the PRP gel (**left**) and CBPGCTS−SF@PRP (**right**) during degradation by enzymes at 0, 24, 48, and 72 h. (**c**,**d**) Angiogenesis induced by (**c**) PRP gel and (**d**) CBPGCTS−SF@PRP implantation into subcutaneous tissue and muscle at 7 d and 14 d. (**e**,**f**) Quantitative data of angiogenesis per field in the PRP gel and CBPGCTS−SF@PRP groups at (**e**) 7 d and (**f**) 14 d (** *p* < 0.01). Reprinted with permission from ref. [46]. Copyright 2020, American Chemical Society.

Several bioactive molecules exhibit poor stability, which inhibit their biological activity in the body. For example, curcumin is a kind of bioactive molecule with antimicrobial, anti-inflammatory and antioxidant abilities, but its poor water solubility leads to low bioavailability [43]. Encapsulating it in hydrogel can prolong its metabolism and further promote its bioavailability. Qu et al. reported a curcumin-loaded chitosan-based self-healing hydrogel which showed excellent antibacterial ability as wound dressing for joints and skin wound healing [44]. Platelet-rich plasma (PRP) which contains various bioactive molecules, such as exosomes, nerve growth factor, and platelet-derived growth factor, can promote wound healing [45]. However, the rapid degradation of these growth factors limits their application in the treatment of chronic wounds. To deal with this, Qian et al. reported a PRP-loaded chitosan-based self-healing hydrogel (CBPGCTS) with the addition of biocompatible and enzymatic hydrolysis-resistant silk fibroin (SF) (CBPGCTS-SF@PRP) (Figure 7a) [46]. Tests of enzymatic degradation in vitro and angiogenesis in vivo were carried out. The bioactive molecules, such as PDGF-BB, NGF and EVs, from PRP gels were monitored in enzyme solutions at 0, 24, 48, and 72 h. The expression of PDGF-BB and NGF in PRP gel proved negative within 24 h and EVs could be barely seen after 48 h, which indicated that bioactive molecules in PRP gels degraded within 48 h (Figure 7b, left). However, the expression of PDGF-BB, NGF and EVs in CBPGCTS-SF@PRP proved positive during 72 h, which suggested that the chitosan-based self-healing hydrogel prolonged the bioactivity of these molecules (Figure 7b, right). Moreover, the histological sections of the PRP gel-implanted group exhibited fewer blood vessels in 7 d and 14 d than the CBPGCTS-SF@PRP group (Figure 7c,d). Quantitative data agreed well with the qualitative results (Figure 7e,f). Therefore, chitosan-based self-healing hydrogel can promote the bioactivity of growth factors and controlled release, making it suitable for chronic diabetic wound healing.

**Figure 8:**
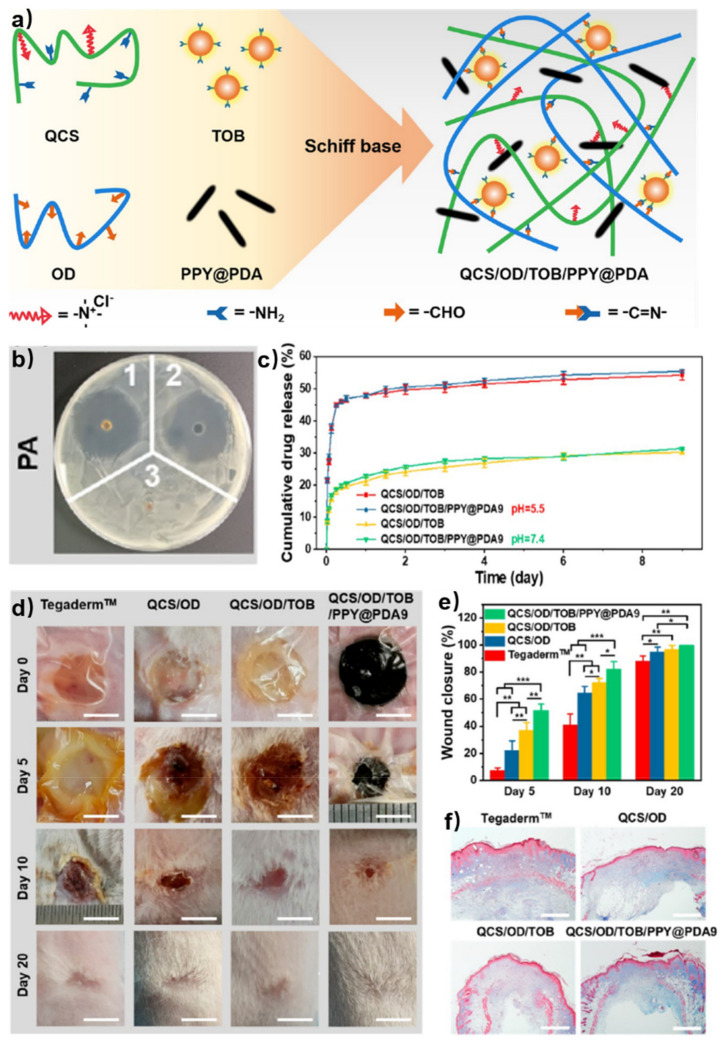
(**a**) Schematic illustration of self-healing hydrogels based on QCS and OD. (**b**) Inhibition zone diameters of PA. (1 for QCS/OD/TOB, 2 for QCS/OD/TOB/PPY@PDA9, and 3 for QCS/OD). (**c**) The release behavior of in QCS/OD/TOB hydrogel and QCS/OD/TOB/PPY@PDA9 hydrogel at pH = 7.4 and pH = 5.5. (**d**) Wound photographs at 0, 5, 10, 20 d for different treatments. Scale bar: 5 mm. (**e**) Wound contraction at 5, 10, 20 d after treatments of different hydrogels. *, **, and *** each represent *p* < 0.05, *p* < 0.01 and *p* < 0.001. (**f**) The collagen deposition at 10 d after treatments of different hydrogels. Scale bar: 800 μm. Reprinted with permission from ref. [47]. Copyright 2022, American Chemical Society.

Smart release of bioactive molecules needs more than physical encapsulation. The dynamic network provides free amino/aldehyde groups, allowing other dynamic connections between additive bioactive molecules and hydrogel networks. Therefore, the bioactive molecules can be controllably released due to stimuli responsive interactions. Huang et al. reported a tobramycin (TOB) smart release self-healing hydrogel for burn wound healing [47]. One of the gel precursors was quaternized chitosan (QCS) with the ability to resist drug-resistant bacteria. Oxidized dextran (OD) with aldehyde groups was selected to form a self-healing hydrogel with QCS via imine bonds. TOB, known as an aminoglycoside antibiotic with rich amino groups, was introduced to the OCS/OD hydrogel through imine bonds to realize the sustained release of TOB. Polydopamine-coated poly-pyrrole (PPY@PDA) nanowires were incorporated to enhance the wound-healing ability of the hydrogel (Figure 8a). The versatility of gel precursors and the tolerance of hydrogels to the embedding of various functional ingredients promote the development of chitosan-based self-healing hydrogels for wound healing. In the agar diffusion test, both QCS/OD/TOB (1 in Figure 8b) and QCS/OD/TOB/PPY@PDA (2 in Figure 8b) exhibited great antibacterial activity for pseudomonas aeruginosa (PA). The release behavior of TOB in vitro was also evaluated. A larger amount of TOB was released in a slightly acidic microenvironment (PBS, pH = 5.5) compared with that in a physiological microenvironment (PBS, pH = 7.4), because imine bonds were fragile to acid. Despite the continuous release of TPB in the physiological microenvironment over 9 days, the amount of TOB release was only 31% (55% in the slightly acidic microenvironment); the slow release of TOB in QCS/OD/TOB/PPY@PDA hydrogels could be attributed to the cross-linking reaction between TOB and OD in the hydrogels (Figure 8c). Then, the wound healing test in vivo using a PA-infected burn wound model was investigated. The QCS/OD/TOB/PPY@PDA hydrogel showed better effect of wound contraction (Figure 8d,e) and a higher amount of collagen deposition (Figure 8f) than the other groups, which indicated excellent ability in promoting the healing of infected burn wounds.

### 3.2. Customized Drug Delivery

Customized drug delivery has been widely used in cancer therapy. Chemotherapy is considered as the primary choice for cancer treatment, where chemotherapy drugs are delivered to the whole body through the blood circulation. However, the lack of drug concentration in lesion sites after circulation needs continuous drug uptake; most chemotherapy drugs carry the risks of both short-term and long-term toxic effects, which cause pain to patients [48]. Chitosan-based self-healing hydrogels are excellent candidates for drug delivery due to the above-mentioned bioactivity of chitosan [49]. Injectability and self-healing ability allow for targeted drug delivery with high concentration, leading to improved utilization efficiency and decreased toxicity to normal tissues.

**Figure 9:**
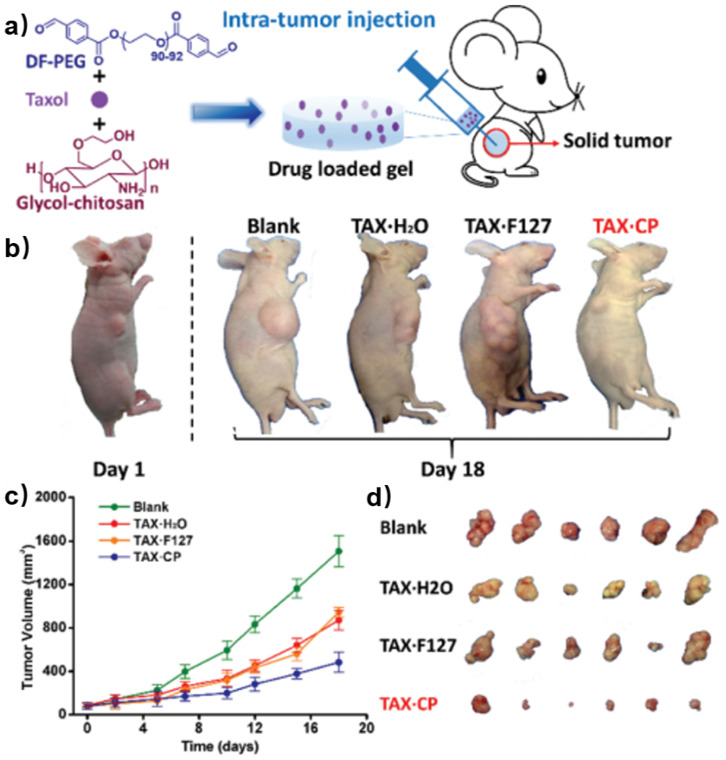
(**a**) Schematic illustration of the intra-tumor injection of Taxol-loaded chitosan-based hydrogel injection (TAX·CP). (**b**) Pictures of nude mice from different groups. (**c**) Tumor volume of mice under different treatments. (**d**) Tumors of the four groups on the 18th day. Reprinted with permission from ref. [52]. Copyright 2017, Royal Society of Chemistry.

Taxol is a well-known natural antitumor drug with poor water solubility [50,51]. Yang et al. encapsulated Taxol into the chitosan-based self-healing hydrogel formed by DF-PEG and GC [52]. The drug-loaded hydrogel was then injected to in vivo intra-tumors of nude mice (Figure 9a). A series of images of the tumor-bearing mice showed that the tumor volumes of the mice under treatment with Taxol solution (TAX·H_2_O) and Taxol-embedded Pluronic F127 hydrogel (TAX·F127) were smaller than those in blank groups. This indicated that Taxol had distinct antitumor ability while F127 hydrogel did not improve the therapy efficacy. However, the mice under treatment with Taxol-loaded chitosan-based self-healing hydrogel (TAX·CP) showed the smallest tumor volume among all groups, demonstrating that the CP hydrogel not only realized targeted drug delivery, but also promoted the antitumor efficacy of Taxol (Figure 9b,d). The quantitative data agreed well with the qualitative results (Figure 9c). Doxorubicin (DOX) is another antitumor drug with a broad spectrum. However, DOX has high cardiotoxicity and drug resistance [53]. In order to diminish the toxicity of DOX and improve therapy efficacy, Qu et al. reported a DOX-loaded chitosan-based self-healing hydrogel which exhibited in vitro pH-responsive gel degradation and DOX release [54]. These results suggested that chitosan-based self-healing hydrogel had great potential to be a drug carrier for hepatocellular carcinoma therapy.

Up to now, chitosan-based self-healing hydrogels have been developed as multifunctional carriers to achieve synergistic efficacy for cancer therapy through physical and chemical interactions. Cancer treatments using optics include photothermal therapy (PTT) [55] and photodynamic therapy (PDT) [56], which are often used alone or in combination with other therapies.

The combination of PTT and tumor proliferation inhibition therapy within chitosan-based hydrogel can improve the precision of cancer treatment. Qi et al. formed a self-healing hydrogel using DF-PEG and CMC [57]. Graphene oxide was carried as a photothermal agent (PTA) and needle-like nano-hydroxyapatite was contained as a tumor inhibitor. This hydrogel effectively inhibited tumor cell proliferation.

**Figure 10:**
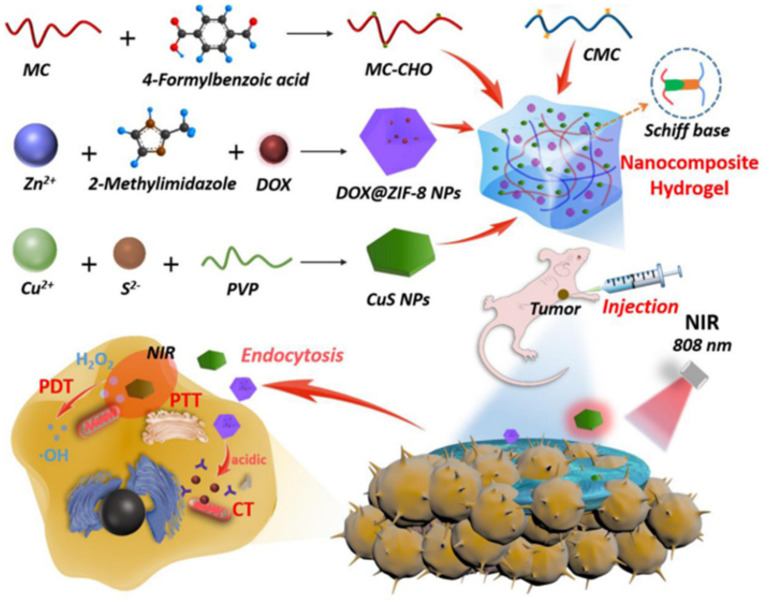
Schematic illustration of the fabrication of MC–CHO/CMC/CuS/DOX@ZIF-8 injectable self-healing nanocomposite hydrogel. Reprinted with permission from ref. [58]. Copyright 2022, Elsevier Ltd.

Moreover, chitosan-based self-healing can efficiently combine drugs, PTA and photosensitizers as a multifunctional carrier to achieve synergistic chemo–photothermal–photodynamic therapy for breast cancer. Qian et al. reported a chitosan-based self-healing hydrogel with aldehyde-modified methylcellulose (MC-CHO) and CMC (Figure 10) [58]. ZIF-8 is a subclass of mental–organic framework, which has high specific surface area for drug loading. Thus, it was selected to combine with DOX to achieve rationally controlled drug release and high drug loading capacity. CuS nanoparticles were included as photothermal agents which can convert light energy into heat energy to trigger protein denaturation of cancer cells. CuS nanoparticles were also reported to generate reactive oxygen species to kill tumors by near-infrared region absorption, thus playing the role of photosensitizers. This hydrogel could be injected directly to the tumor site for treatment, owing to the dynamic imine network.

### 3.3. Smart Biosensors

Chitosan-based self-healing hydrogels are biocompatible, flexible and responsive to environmental changes, and offer great potential for biosensing. Moreover, considering that chitosan is a cationic polymer with possible conductivity, many researchers have been focusing on chitosan-based self-healing hydrogels as materials for smart biosensors, which can convert external stimuli change (strain variations et al.) to electrical or optical signals change for applications in wearable electronic devices, human motion detection, health monitoring, and artificial smart skin [59,60].

Wang et al. designed an optical glucose biosensor using glucose-sensitive hydrogel films as both glucose-sensing material and Fabry–Perot cavity [61]. The film was prepared by layer-by-layer assembly from partially oxidized dextran (PO-Dex), chitosan, and glucose oxidase (GOD). The GOD can convert glucose to gluconic acid, leading to the decrease in pH. Then, the chitosan-based self-healing hydrogel film swells because the dynamic imine bonds are sensitive to low pH. The swelling induced by glucose leads to a shift of Fabry–Perot fringes on the reflection spectra of the film, enabling the determination of glucose concentration. Besides optical signals, electrical signals can also be detected by incorporating conductive substances into the hydrogels. Liang et al. covalently linked CeO_2_/MnO_2_ hollow nanospheres to a self-healing hydrogel based on QCS and OD [62]. GOD was loaded by the strong electrostatic interactions with modified CeO_2_/MnO_2_ hollow nanospheres to avoid leakage. The flexible glucose sensor showed a wide linear range (1–111 mM), fast response (less than 3 s) and high sensitivity (176 μA mM^−1^ cm^−2^); the self-healing ability endowed the hydrogel with the ability to work continuously for over 30 days.

**Figure 11:**
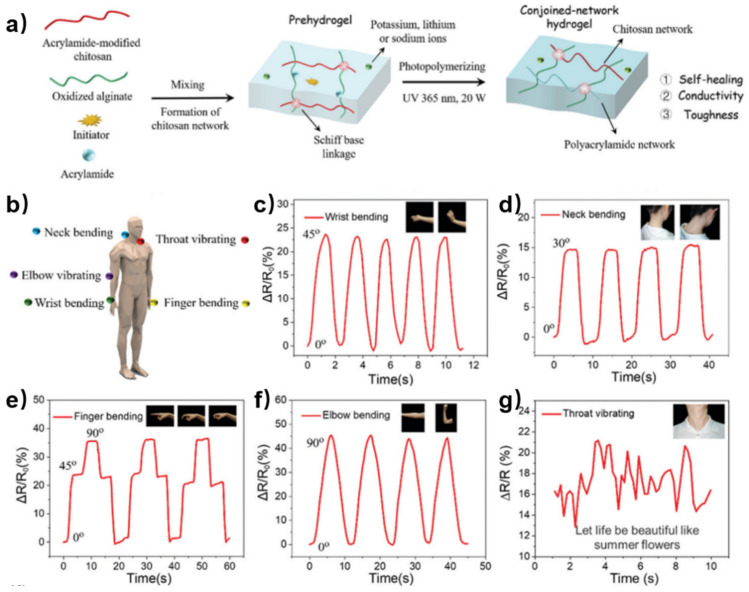
(**a**) Schematic illustration of the preparation of the conjoined-network hydrogels. (**b**) Schematic illustration of the hydrogel sensor attached to various parts of the human body. (**c**–**g**) Relative resistance changes in the hydrogel sensor attached to different parts of the human body (wrist, neck, finger, elbow, and throat) Reprinted with permission from ref. [63]. Copyright 2022, Royal Society of Chemistry.

Incorporating another polymer network into chitosan-based self-healing hydrogel can enhance the mechanical performance of biosensors. Ding et al. reported a robust self-healing hydrogel through acrylamide-modified chitosan, oxidized alginate and polyacrylamide [63]. The acrylamide-modified chitosan and oxidized alginate formed the first network by dynamic imine bonds and hydrogen bonding. Then, free and branched acrylamide photopolymerized in the chitosan hydrogel network to form the second permanent network (Figure 11a). Free ions in the hydrogel provided ionic conductivity. Owing to the self-healing ability, high toughness and ionic conductivity, this hydrogel was fabricated as a strain sensor for motion detection (Figure 11b). The sensor can precisely monitor the movement of the wrist (Figure 11c), neck (Figure 11d), finger (Figure 11e), elbow (Figure 11f), and throat (Figure 11g) with repeatable electrical signals, indicating the wide application of the hydrogel sensor.

### 3.4. 3D/4D Printing

3D printing is a promising technique to fabricate personalized biomedical devices with precisely controllable shapes and delicate structures. Chitosan-based self-healing hydrogel is a candidate biomaterial for 3D printing due to its excellent injectability, biocompatibility and controllable mobility. Li et al. designed a mesenchymal stem cells-loaded self-healing hydrogel based on hydroxy-butyl chitosan and oxidized chondroitin sulfate [64]. The hydrogel was printed using a sacrificial mold. The 3D printed hydrogel scaffold exhibited good formability and biocompatibility, which showed great potential for cartilage tissue engineering.

**Figure 12:**
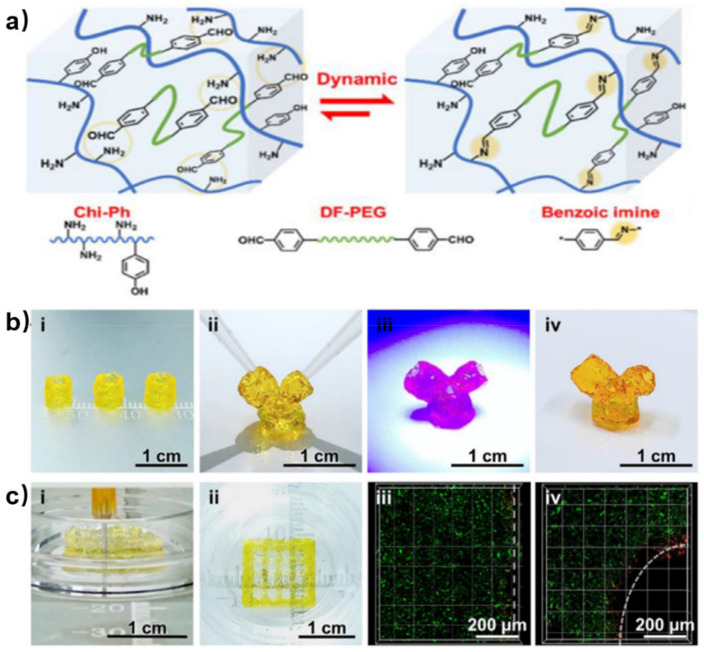
(**a**) Schematic illustration for preparation of chitosan-based self-healing hydrogel by dynamic benzoic imine crosslinking between Chi-Ph and DF-PEG. (**b**) Assembling of modular 3D printed hydrogel constructs. (**c**) 3D bioprinting of cell-laden photo-cross-linkable hydrogel. Reprinted with permission from ref. [65]. Copyright 2021, Elsevier Ltd.

Stable rheological properties and post-printing crosslinking are important for self-healing hydrogels used in 3D printing. Liu et al. designed a self-healing hydrogel using DF-PEG and phenol-functionalized chitosan (Chi-Ph) which can be crosslinked by visible light (Figure 12a) [65]. The first network was made by dynamic imine bonds, which endowed the hydrogel with excellent self-healing ability and fast gelation. The second network was attributed to the visible light-crosslinking of irreversible phenol–phenol bonds between Chi-Ph chains. The visible light-triggered post-printing could provide a strengthened network with better cell compatibility compared with UV-triggered post-printing [66]. In this work, the chitosan-based self-healing hydrogel was modularly printed as a 3D structure; three tube-like building blocks were assembled in a “Y” shape. Then, blue light was applied to strengthen the “Y”-like hydrogel by secondary crosslinking which could keep the structure stable after violent shaking (Figure 12b). More sophisticated lattice structure was 3D-bioprinted. The loaded cell showed high survivability according to the results of live/dead staining (Figure 12c). However, the printing resolution and biostability of the self-healing hydrogel need to be further improved for 3D bioprinting.

**Figure 13:**
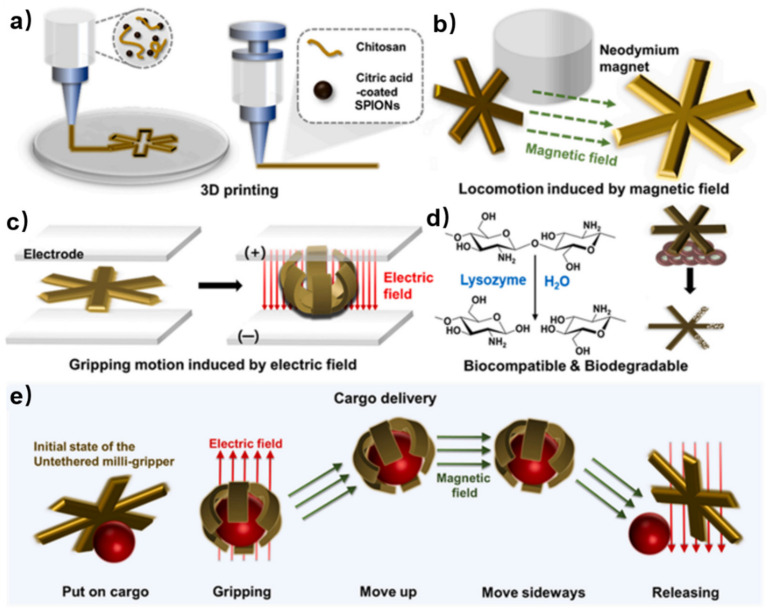
(**a**) 3D printing of the untethered milli-gripper composed of an ink containing the chitosan hydrogel and citric acid-coated SPIONs. (**b**) Motion of the untethered milli-gripper induced by a magnetic field generated using a neodymium permanent magnet. (**c**) Gripping motion of the untethered milli-gripper induced by an electric field. (**d**) Biocompatibility and biodegradability of the untethered milli-gripper. (**e**) Overall process of cargo delivery. Reprinted with permission from ref. [67]. Copyright 2023, Elsevier Ltd.

While 3D printing focuses on creating three-dimensional objects layer by layer, 4D printing takes this a step further by adding the dimension of time, allowing the printed objects to transform or self-assemble into predetermined shapes or functionalities. 4D printing of chitosan-based self-healing hydrogels enables the fabrication of medical soft robots, which require biocompatibility and biodegradability. Jang et al. fabricated a 4D printed untethered milli-gripper using a 3D printer and an ink composed of a biocompatible and biodegradable chitosan hydrogel [67]. This ink was supplemented with citric acid-coated superparamagnetic iron oxide nanoparticles (SPIONs) which allows for shape change of the milli-gripper after printing (Figure 13a). The hydrogel ink was self-healing due to the electrostatic interactions between chitosan and citric acid. By incorporating citric acid-coated SPIONs, the milli-gripper can move to an accurate position in 3D space using a neodymium permanent magnet (Figure 13b). When an electrolyte is subjected to an applied electric field, the untethered milli-gripper experiences deswelling towards the positive electrode (anode). This deswelling induces bending of the milli-gripper towards the anode, enabling gripping motion (Figure 13c). Moreover, used in the body, the chitosan part undergoes biodegradation via lysozyme, while the biocompatible citric acid-coated SPIONs are excreted and do not accumulate in the body (Figure 13d). Thus, the untethered milli-gripper can grasp cargo which is driven by the electric field and transport it to the desired place under the influence of the magnetic field. The cargo can be released at the target point by reapplying an electric field (Figure 13e). Moreover, the shape of chitosan-based self-healing hydrogels after 3D printing can also be changed by solvent trigger, showing potential as a medical robot [68].

## 4. Summary and Outlook

Self-healing hydrogels constructed by physical interactions or dynamic covalent bonds have been attractive materials for biological applications. Among them, chitosan-based self-healing hydrogels stand out due to their mild preparation conditions, excellent biocompatibility, and self-recovery ability under physiological environment. The self-healing properties (e.g., self-healing speed) can be influenced by different aldehyde crosslinkers, crosslinking density and external conditions. In this review, we summarize the design and fabrication of chitosan-based self-healing hydrogels, mainly focusing on the development of aldehyde-modified crosslinkers. Furthermore, we present the biological applications of chitosan-based self-healing hydrogels in tissue regeneration, customized drug delivery, smart biosensors, and 3D/4D printing, where the hydrogels played a role as excellent carriers.

However, there remain several challenges in the development of chitosan-based self-healing hydrogels. For example, DF-PEG has been widely used for hydrogel construction. The molecular weight of PEG for the synthesis of DF-PEG is important. PEG with low molecular weight has great reactive activity, but it may have toxicity concerns for biological use; PEG with high molecular weight improves biosafety, but it introduces challenges in synthesis. Thus, the range of available PEG is limited. Moreover, the aldehyde-functionalized PEG merely plays the role as a gel precursor in many research projects, limiting its applications as an intrinsic functional material. To solve this problem, developing new methods to prepare gel precursors with available aldehyde groups and other functions may be a good alternative to DF-PEG. For example, mild reaction conditions should be applied to modify biocompatible polymers which contain reactive groups to bestow them with aldehyde groups. In addition, more effort should be made to investigate the toxic effects, immunogenicity and fabrication challenge of the additional molecules that are used for enhancing the chemical properties of chitosan.

Furthermore, chitosan-based self-healing hydrogel is tolerant to many functional building blocks as a great carrier, so it is fascinating to develop multicomponent self-healing hydrogel with various functions. By combination with artificial intelligence for deep learning, multifunctional hydrogels hold great promise in applications in cutting-edge fields, such as smart diagnosis and treatment.

## Data Availability

The data are available from the authors of the articles’.

## Abbreviations

3D/4D	Three/four dimensional
ECM	Extracellular matrix
PEG	Polyethylene glycol
DF-PEG	Dibenzaldehyde-terminated telechelic PEG
PEG-BA	Benzaldehyde-terminated telechelic four-armed PEG
CMC	Carboxymethyl chitosan
GC	Glycol chitosan
MC	Methylcellulose
DAMC	Dialdehyde form of MC
CHI-O	Chitosan oligomer
NSCs	Neural stem cells
HA	Hyaluronan
SIPN	Semi-interpenetrating polymer network
PRP	Platelet-rich plasma
SF	Silk fibroin
TOB	Tobramycin
QCS	Quaternized chitosan
OD	Oxidized dextran
PPY	Polypyrrole
PDA	Polydopamine
PA	Pseudomonas aeruginosa
DOX	Doxorubicin
PTT	Photothermal therapy
PDT	Photodynamic therapy
PTA	Photothermal agent
MC-CHO	Aldehyde-modified methylcellulose
PO-Dex	Partially oxidized dextran
GOD	Glucose oxidase
Chi-Ph	Phenol-functionalized chitosan
SPIONs	Superparamagnetic iron oxide nanoparticles
